# Infradian rhythms of human heart rate reveal distinct multiday cycle periods in healthy adults

**DOI:** 10.1016/j.ebiom.2026.106444

**Published:** 2026-08-22

**Authors:** Rochelle I. De Silva, Rachel E. Stirling, Jodie Naim-Feil, Shivam Puri, Elizabeth Paratz, Philippa J. Karoly

**Affiliations:** a Department of Biomedical Engineering, University of Melbourne, Melbourne, Australia; b Graeme Clark Institute, University of Melbourne, Melbourne, Australia; c Department of Medicine, St. Vincent’s Hospital Melbourne, University of Melbourne, Melbourne, Australia

**Keywords:** Infradian, Biological rhythms, Cardiac, Autonomic, Wearables

## Abstract

**Background:**

Chronobiology research has historically focused on circadian rhythms; however, longer infradian rhythms are prevalent in human physiology and may have important implications for health and wellbeing. Previous studies have identified widespread infradian rhythms across human physiology, often in the context of hormonal regulation and disease. Despite growing evidence of their ubiquity, the mechanisms, significance, and clinical relevance of these rhythms remain poorly understood, largely reflecting a lack of longitudinal datasets and robust detection methods. The emergence of new wearable technologies enables rich, continuous data capture within individuals, allowing physiological rhythms to be studied at scale.

**Methods:**

This retrospective, observational cohort study analysed young adults (n = 623), with up to four years of wearable and questionnaire data collected through the University of Notre Dame’s (USA) NetHealth project. Participants who recorded at least three months of continuous (>80% data completeness) heart rate data were included and significant infradian rhythms were identified using wavelet analysis. Unsupervised non-negative matrix factorisation was performed to cluster similar wavelet power spectrum distributions. Individuals’ heart rate rhythms were compared to known environmental cycles (day-of-week, lunar, seasonal) and considering demographics and social networks. A second, smaller cohort (n = 70) with heart rate and menstrual timing was included to analyse the interplay of hormonal regulation on monthly cycles. Multinomial logistic regression and the Kruskal–Wallis test were applied to quantify the effects of environmental, behavioural and demographic factors on heart rate rhythms.

**Findings:**

Significant infradian rhythms of heart rate were detected in 70% (369/525) of the cohort and 36% (187/525) had two or more rhythms. Annual, biannual and 10-week rhythms were the most common. Within the 4–45-day band, individuals clustered into three multiday chronotypes based on dominant periodicities in their wavelet power spectra: weekly (∼7 days), monthly (∼25–35 days), and multi-month (>35 days). Heart rate rhythms were influenced by environmental cycles (day-of-week and seasonality) and were synchronised to the menstrual cycle in most menstruating females, although monthly rhythms were also observed in males and menopausal women.

**Interpretation:**

The prevalence of infradian, or multiday heart rate rhythms in healthy young people motivates further scientific investigation to understand the mechanisms of these rhythms and their potential association with autonomic function, and risk of disease or disease-specific symptoms.

**Funding:**

This study was funded by an 10.13039/501100000923Australian Research Council Grant (DP240102899). This study also received funding from the Australian National Health and Medical Research Council Medical Research Future Fund (MRFF 2039966).

**Research in context**

**Evidence before this study**

Before conducting this study, we searched the literature for any studies that investigated infradian rhythms in humans, predominately in the areas of autonomic physiology and cardiology. We used Google Scholar and PubMed with search strategies that combined one or more of the terms; “infradian”, “rhythm”, “multiday”, “circaseptan”, “weekly”, “monthly”, “lunar”, “circatrigintan”, “seasonal”, “heart”, “heart rate”, “cardiac”, “arrhythmia”, “blood pressure”, with no restrictions on language or dates. A few older texts and case studies observed infradian rhythms in the heart rate or blood pressure of individuals at multiday periodicities, particularly weekly and seasonal cycles which were thought to have environmental origin. Multiday patterns in the occurrence of cardiac abnormalities including arrhythmia and heart attacks had also been confirmed in recent studies, predominantly considering day-of-week and lunar or seasonal effects at the population-level. Female menstrual cycles were commonly correlated to rhythmic fluctuations in autonomic biomarkers, including wearable-recorded heart rate. However, other long-term, individual rhythms of cardiac activity outside of known environmental zeitgebers were rarely considered. One cohort study assessed continuous implant recordings from people with cardiovascular disease and found weekly, quarterly (3-month) and seasonal cycles at the individual level. Another study showed seven-day periodicities in the heart rate of neonates. Longitudinal infradian rhythms of heart rate, and temperature from wearable data were linked to seizures for some adults with epilepsy and a small group (n = 15) of control participants also showed multiday heart rate cycles. There is limited investigation of multiday heart rate cycles over long timeframes in the wider population.

**Added value of this study**

We utilised a large cohort (n = 623) of college-aged adults enrolled in an observational study, NetHealth, which captured wearable heart rate for 2–4 years per person. This allowed us to identify heart rate cycles over multiday time scales and consider the effects of demographic (sex), behavioural (activity, sleep, social networks) and environmental factors (day-of-week, lunar phase, season) as drivers of infradian rhythms in the heart.

**Implications of all the available evidence**

Prominent, individual-specific infradian heart rate rhythms were identified in 70% (369/525) of people, with clusters identified at weekly (∼7 days), monthly (∼25–35 days), and multi-month (>35 days). Longer, biannual and annual cycles were also common. Heart rate rhythms were influenced by environmental cycles (day-of-week and seasonality) but were not tightly correlated to external cues. Additionally, heart rate rhythms were synchronised to the menstrual cycle in most menstruating females, although monthly rhythms were also observed in males and menopausal women.

Heart rate rhythms may have applications in modelling disease; for instance, in epilepsy these rhythms can provide a biomarker for individual seizure likelihood. The broader potential for heart rate rhythms to herald vulnerabilities in related autonomic systems including individual susceptibility to stress, mood fluctuations or other disease symptoms warrants investigation. More generally, a better understanding of these systemic, physiological cycles may shed new light on human biological rhythms and disease.

## Introduction

Circadian and longer, infradian rhythms are pervasive in the natural world, shaping reproduction, development, activity and behaviour across marine and terrestrial organisms. Circadian biological rhythms are well-documented in humans and other organisms and are entrained by the external environment via light–dark cues from the solar day. These rhythms can persist in the absence of environmental cues (i.e., free-running) driven by endogenous, self-sustained oscillators. Unlike circadian rhythms, longer biological rhythms remain less widely characterised; however, infradian rhythms in humans have been observed across timescales, including weekly (7-day),^1^, ^2^, ^3^ monthly (28–31-days),^4^, ^5^, ^6^ and seasonal (yearly).^7^, ^8^, ^9^ Examples of these infradian rhythms include mammalian growth cycles of enamel and bone deposition,^10^^,^^11^ human heart rate,^12^^,^^13^ and blood pressure.^2^^,^^3^^,^^14^ Moreover, studies conducted in isolation report longer rhythms in heart rate, blood pressure, and hormone secretion can persist in the absence of zeitgebers (i.e., external environmental cues).^1^^,^^2^ One study documented 7-day rhythms in heart rate and blood pressure in a woman living in social isolation over a 14-week period.^2^ More recent studies in cohorts with chronic brain implants found multiday (or ‘multidien’) rhythms of epileptic activity independent from environmental cues.^15^, ^16^, ^17^ Although individual studies remain limited in size or scope, collectively there is an emerging body of evidence across immunology,^18^ cardiology,^13^^,^^19^ and neurology^12^ showing longer rhythms in humans that appear to follow free-running cycles with periods from weeks to months.

Several mechanisms have been proposed as potential drivers of infradian rhythms observed in humans. While fixed calendar-based (e.g., weekday^1^) or environmental (e.g., lunar) cycles^6^ may explain some observations, longer rhythms are also hypothesised to arise from internal mechanisms, including, hormonal fluctuations,^8^^,^^9^^,^^20^ immune system fluctuations,^18^^,^^21^ metabolic processes,^22^^,^^23^ or autonomic regulation.^12^ The menstrual cycle often dominates perceptions of monthly rhythms; yet, men can also exhibit cyclical patterns on a monthly scale.^4^^,^^12^ This is particularly evident in neurological cohorts where similar monthly fluctuations in brain and seizure activity are observed for men, women and children.^24^^,^^25^ Furthermore, neurological rhythms have individual-specific periods ranging from weekly to seasonal^24^; therefore, mechanistic theories targeted at one cycle length cannot account for the striking prevalence nor heterogeneity of rhythms observed across central and autonomic nervous activity. Indeed, 60% of adults with epilepsy show infradian rhythms of brain activity, with similar proportions having weekly, fortnightly, monthly or longer cycles.^26^ Outside the brain, a study of over 300 patients with cardiovascular disease found many had annual (78%), 7-day (26%) and 3-monthly (22%) rhythms in heart rate, although monthly cycles were rare.^13^ A smaller cohort study using wearables found weekly and monthly heart rate rhythms were common in healthy adults.^12^ Despite increasing evidence that infradian rhythms are a feature of human brain and heart activity, the prevalence of these rhythms in the wider population is not well established and consequently their underlying mechanisms, functional significance, and clinical relevance remain largely unknown.

Recent technological advances to enable continuous physiological monitoring and large-scale population data provide an opportunity to address whether infradian rhythms are endogenous or environmental. Wearable signals to track peripheral oscillatory systems are increasingly validated as proxies for less accessible central or hormonal oscillatory dynamics,^27^^,^^28^ and offer longitudinal insights into coupled physiological rhythms between the heart and brain.^12^^,^^29^ This study aimed to investigate long-term rhythms of heart rate in healthy adults, characterising their prevalence, heterogeneity, and environmental influences, using a large database of wearable recordings.^30^ The majority had heart rate rhythms with cycle periods ranging from weekly, monthly to longer multi-month or seasonal periods. Establishing normative patterns on infradian rhythms in healthy adults is a critical step toward identifying their governing drivers and understanding how multiday fluctuations can arise in disease symptoms across neurological,^15^^,^^24^ psychiatric,^20^^,^^31^ and broader health conditions. Advancing this understanding could reshape current models of physiology and drive applications in disease prediction, monitoring, and treatment.

## Methods

The primary objective of the study was to assess the presence of infradian rhythms in resting heart rate. Secondary objectives included the relationship of infradian heart rate rhythms with demographic, behavioural, physiological, and environmental factors. The study outcomes were derived from post-hoc analyses of retrospective, publicly available data from longitudinal, observational cohorts. Sample-size determination was not applicable to this retrospective analyses. The original studies were non-interventional and did not conduct randomisation or blinding.

### Dataset and preprocessing

#### NetHealth dataset

This study analysed data from the University of Notre Dame’s NetHealth project^32^ (Indiana, U.S.A.), a publicly available longitudinal dataset that followed a cohort of university students over four years (August 2015–May 2019). Participants (n = 623) wore Fitbit smartwatches (either Charge HR or Charge 2 model), which continuously recorded health-related metrics, including heart rate, physical activity and sleep.^30^ The individual wearable device data included timestamped heart rate recorded from the smartwatch (in beats per minute), as well as minutes of daily physical activity (defined by Fitbit metabolic equivalent tasks (MET) model, i.e., 1.5–3.0 METs categorised as ‘lightly active minutes’, 3.0–6.0 METs for ‘fairly active minutes’, and >6.0 METs for ‘very active minutes’) and daily sleep metrics (including estimated sleep time, wake time and duration).

In addition to the wearable data, this study incorporated selected survey measures and demographic data available within the NetHealth dataset. Demographic data included age and sex, both of which were self-reported. Surveys included the Morningness–Eveningness Questionnaire (MEQ), which assessed self-reported circadian preference or chronotype, and a Social Network survey, in which individuals in the cohort reported the 20 other individuals they spent the most time with over the previous few months (often containing other participants in the study). A summary of the participant demographics and characteristics can be found in Supplementary Table S1. The dataset and accompanying documentation are accessible at https://sites.nd.edu/nethealth/.

This study used individual heart rate data per minute to identify infradian rhythms. First, the minute-level heart rate data were pre-processed to derive an approximation of daily resting heart rate. Heart rate was resampled to daily values by taking an average across the lowest 30th percentile of heart rate for each day (24-h period from midnight to midnight). This proxy resting heart rate, rather than daily average heart rate, was chosen to reduce the potential impact of physical activity, which was correlated with average heart rate (Supplementary Appendix 1).

Participants with at least 3 months of recorded heart rate were included in the analysis (study flow diagrams can be found in Supplementary Appendix 2). Spectral analysis was conducted on the highest-quality data segment, defined as the longest continuous segment (of at least 3 months or 91.3-days) with fewer than 20% missing data points (equivalent to a cumulative maximum of 18.2 days missing for an exact 3-month period). Missing segments were interpolated with the global average (validated in previous work^12^). Briefly, simulation analyses comparing time-matched imputation strategies (including filling from matched time-of-day averages and matched prior-day values) to global mean imputation demonstrated that global mean imputation was the most effective in determining true peaks in the periodogram while minimising the detection of false peaks. To confirm robustness of our findings to missing data, a supplementary analysis (Supplementary Appendix 3) was conducted restricting analyses to segments with no more than 5 consecutive days of missing data.

#### Soochow dataset

To investigate the influence of the menstrual cycle on infradian rhythms of heart rate, this study also analysed longitudinal wearable and menstrual cycle data captured between November 2017 and October 2020 as part of a study by Soochow University, China.^33^ This publicly available dataset included 91 menstruating females (mean age = 23.52 ± 2.81 years), 12 postmenopausal females (age = 57.08 ± 5.44) and 15 males (age = 38.80 ± 10.82) who wore Huawei or Fitbit smartwatch devices capturing minute-level heart rate data. The mean recording duration was 368.46 days (standard deviation, SD = 252.75 days). Within the menstruating females cohort, 84 tracked menstrual periods and 28 additionally recorded ovulation using luteinising hormone strips. Ovulation phase was defined as the fertile window (three days prior to ovulation, ovulation day, and one day after); when ovulation was not recorded, it was estimated as the five-day window immediately preceding the luteal phase (i.e., 14 days before menstruation).

The minute-level heart rate data were used to estimate daily resting heart rate and analysed in the same way as the NetHealth dataset to extract multiday rhythms (details in *Multiday rhythm estimation* section). Only participants with at least 3 months of continuous recorded heart rate (defined as greater than 80% data completeness) were included in the analysis.

### Multiday rhythm estimation

Heart rate data were analysed using a wavelet decomposition method with a continuous Morlet wavelet transform, which is well suited to almost-periodic or non-stationary time series^34^ and has previously been applied to detect infradian heart rate rhythms.^12^^,^^15^ Individuals’ processed heart rate segments were z-standardised (by subtracting the mean and dividing by the standard deviation) prior to wavelet analysis. Resulting spectrograms were averaged across time to generate the global wavelet power or periodogram. The global wavelet spectrum or power ${\bar{W}}_{n}^{2}\left(s\right)$ is defined as the time-averaged wavelet power at each frequency.

$${\bar{W}}_{n}^{2}\left(s\right)=\frac{1}{N}\sum_{n=0}^{N-1}{\left|W_{n}\left(s\right)\right|}^{2}$$

where ${\left|W_{n}\left(s\right)\right|}^{2}$ signifies the wavelet power at frequency s and time index n. The global wavelet spectrum is found by averaging the wavelet power across time for each frequency. To determine significant cycles for individuals, a 95% significance threshold (α < 0.05) was applied to the global wavelet periodogram using the time-averaged significance test against a red noise background spectrum described in previous work.^34^ Any peaks in the global wavelet spectrum surpassing this threshold were deemed statistically significant and interpreted as evidence of a rhythmic component in the data and were thus included in subsequent analyses.

Candidate cycle periods ranged from 4 days to a maximum of one-third of an individual’s recording length (i.e., a minimum of three cycles had to be observed) with a step size of 12 h. Restricting the range of periods analysed helped reduce boundary (edge) effects in the wavelet transform, thereby improving reliability of the spectral estimates.

**Fig. 1:**
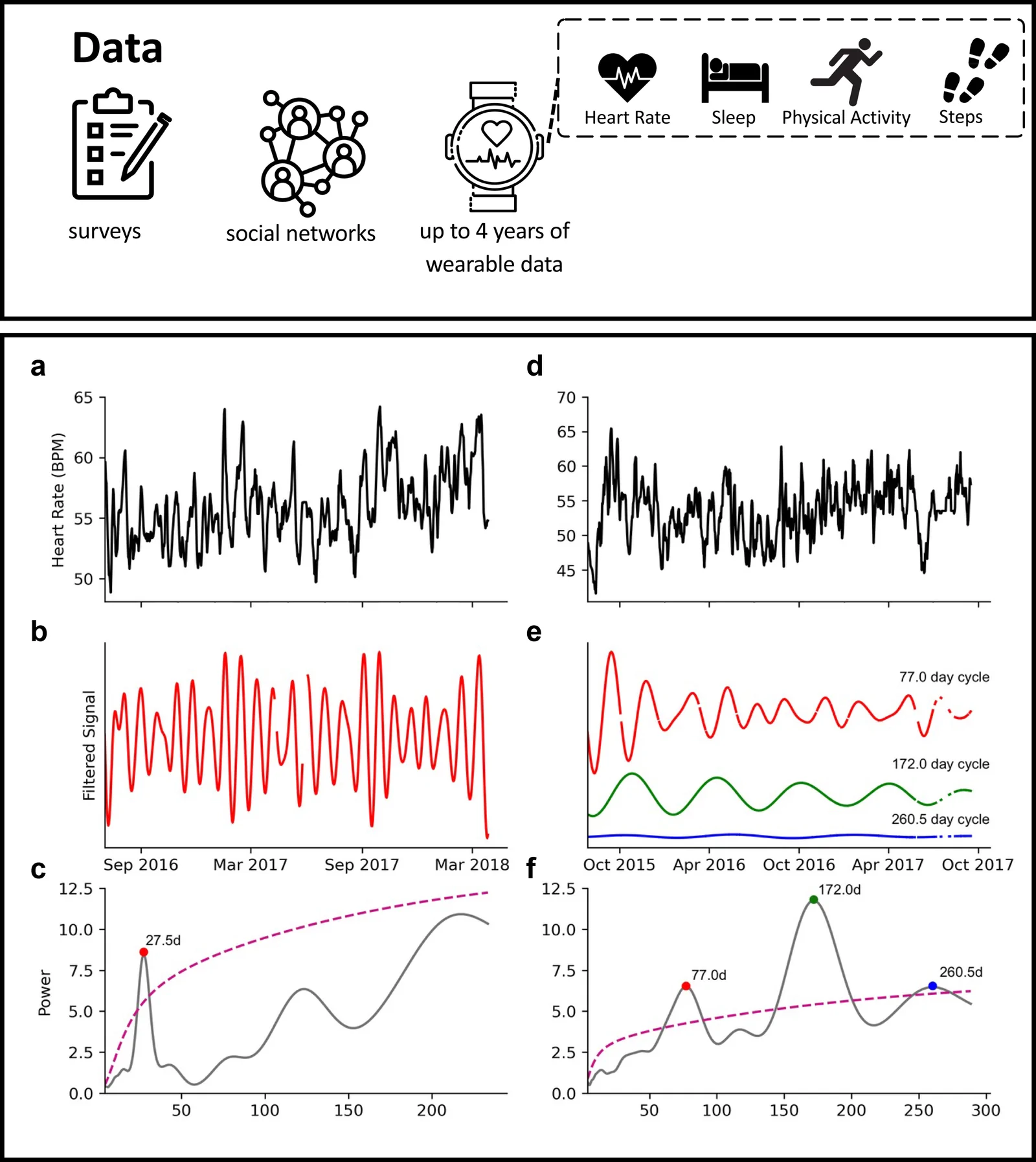
**Schematic of the preprocessing and multiday cycle detection steps.** Survey, social network and wearable smartwatch data (up to 4 years) were provided in the NetHealth project dataset. Heart rate data and multiday cycles are shown for two different individuals: example 1 **(a–c)** and example 2 **(d–f)**. **a,d**: Daily resting heart rate (y-axis) in beats per minute, smoothed with a 4-day moving average filter, shown over 2 years. **b,e**: A graphical representation of the significant multiday cycles identified in the wavelet periodogram. The original daily resting heart rate signal was bandpass filtered to produce the representation, with the passband including frequencies within the full-width-half-maximum of the corresponding peak. **c,f**: Wavelet periodogram for each example at different scales (x-axis). Significant cycle periods (distinct peaks) are labelled with coloured dots.

Each significant peak in the periodogram was defined by its prominence, i.e., the extent to which a peak stands out from the local baseline, measured as the vertical distance between the peak and the surrounding local minima in the periodogram. When more than one significant peak was detected, peaks were assessed for distinctness to avoid counting overlapping peaks in the periodogram as separate cycles. Peaks were considered distinct if they fell outside the full-width-half-maximum range of a neighbouring, more prominent peak. Thus, individuals’ multiday cycles were defined as all statistically significant (i.e., passing the 95% significance threshold), distinct peaks in the periodogram, with the most *dominant* cycle defined as the most prominent peak. Fig. 1 shows recordings from two participants, with significant rhythms in their daily heart rate, detected using wavelet analysis.

### Multiday chronotypes

The concept of multiday chronotypes has previously been proposed as an unsupervised approach to classify common cycle periods across a population of people. For instance, in neurological cohorts, seizure cycles are commonly observed at about-weekly, about-monthly and seasonal periods.^24^ This study used non-negative matrix factorisation (NMF) to quantitatively describe the cyclical trends and clusters across the population without pre-defining set chronotypes. NMF factorises a matrix of averaged power values across periods in each individual’s dataset into two non-negative matrices: weights and components. The optimal number of components was chosen by using a cross-correlation metric described in the supplement (Supplementary Appendix 4). Thus, for each individual, the strongest weight component in the NMF can be identified and this allows sorting of all individuals into chronotypes based on their dominant heart rate cycles.

### Ethics

The datasets analysed during the current study were accessed from two publicly available sources, University of Notre Dame’s NetHealth repository (https://sites.nd.edu/nethealth/), and Soochow University Mendeley repository (https://doi.org/10.17632/v58stpfcnm.1), and contained no personal identifiers.

The NetHealth data collection (August 2015–May 2018) was approved by the University of Notre Dame’s (USA) institutional review board after a full board review under protocol number 17-05-3912. All participants provided written informed consent prior to taking part in the study.

The Soochow University data collection (November 2017–December 2020) was approved by the Ethics Committee of Soochow University (China) under protocol number ECSU-201800098. All subjects provided written consent before taking part in the study and the investigation conformed with the principles outlined in the 2024 Declaration of Helsinki.

### Statistics

#### Environmental effects

The influence of three environmental variables on heart rate rhythms was investigated: day of the week (Monday to Sunday), season (spring, summer, autumn or winter) and lunar phase (coarsely defined into four equal tranches of the 29.53-day synodic cycle, centred around the phases: new moon, waxing, full moon, waning). Daily resting heart rate was standardised (z-scored within individuals) and mapped to environmental cycle phases, and individuals’ mean values were calculated for each environmental cycle phase. This analysis was conducted on the whole cohort and on individuals whose dominant rhythm was weekly (5–9 days), monthly (25–35 days) or annual (11–13 months), to examine whether environmental influences on heart rate were more pronounced in individuals whose dominant rhythm period most closely matched the environmental cycle. For both analyses, a Kruskal–Wallis test was used to assess differences in each comparison group.

A complementary continuous approach was also used to quantify environmental effects on individuals’ heart rate. To isolate individual-specific heart rate rhythmicity from the environmental drivers, a Generalised Additive Model (GAM) was fitted separately to each individual’s standardised resting heart rate time series:

$$z_{HR}\left(t\right)=f_{0}\left(t\right)+f_{1}\left(t,day\;of\;week\right)+f_{2}\left(t,lunar\;phase\right)+f_{3}\left(t,season\right)+\epsilon$$

Where $f_{0}$ captures any varying baseline trend in heart rate over the recording period (modelled as a penalised spline), $f_{1}$, $f_{2}$ and $f_{3}$ are factor-smooth terms (i.e., environmental effects) that allow the contribution of each environmental variable to vary smoothly over time and ϵ is the residual error. The summed contributions of the three environmental factor-smooth terms $\left(f_{1}+f_{2}+f_{3}\right)$ were subtracted from each individual’s standardised heart rate, yielding an environmentally adjusted signal, and used as inputs for a subsequent multiday cycle detection analysis. This enabled the identification of individual multiday cycles that were presumed to be uncorrelated with the 7-day week, lunar, or seasonal cycle.

#### Menstrual cycles

The relationship between the menstrual cycle and resting heart rate was investigated at both a cohort level and an individual level using the Soochow University dataset. On a cohort level, daily resting heart rate was standardised and mapped to menstrual cycle phases (i.e., menstruation, follicular, ovulation, luteal); mean standardised resting heart rate values were calculated for each menstrual cycle phase. To characterise the heart rate modulation across the cycle, a linearised cosinor model was fitted to each individual’s data:

$$z_{HR}=\beta_{0}+\beta_{1}\;\cos\;\left(\frac{2\pi t}{T}\right)+\beta_{2}\;\sin\;\left(\frac{2\pi t}{T}\right)$$

Where T = 28 days and the time variable t was assigned as the actual elapsed day within each cycle, normalised to a 28-day period. Cycles shorter than 15 days or longer than 50 days were excluded as likely recording artefacts, as was the final incomplete cycle per individual. Whilst individual cycle lengths vary, the cosinor fit is applied to phase-averaged heart rate values, meaning the model captures the shape and timing of the rhythm relative to the cycle structure rather than relying on the period. Significance was assessed using an F-test comparing the full cosinor model against an intercept-only null model, F(2,n−3), with a threshold of p < 0.05. The amplitude and acrophase (day of peak) were derived from the fitted β coefficients as amplitude = $\surd \left(\beta_{1}^{2}+\beta_{2}^{2}\right)$ and acrophase = $\mathrm{atan}\;2\left({-\beta }_{2},\beta_{1}\right)$. The cohort mean cosinor curve was computed by averaging the β coefficients across individuals with a significant fit.

At the individual level, the synchronisation between menstrual days compared to the individual’s dominant heart rate rhythm (any duration in days) and lunar phase (29.53-day synodic cycle) was quantified using the corrected synchronisation index (SI)^35^:

$$SI\left(T\right)=\frac{\left|\frac{1}{N}\sum_{n=0}^{N}e^{i\theta \left(T,n\right)}\right|-\gamma_{N}\;}{\left(1-\gamma_{N}\right)}$$

where N is the total number of reported menstruation periods, θ(T,n) is the cycle phase at menses onset of the n^th^ menstruation period relative to the cycle period T, i is the imaginary number and $\gamma_{N}$ is the correction factor:

$$\gamma_{N}=\frac{1}{2}\sqrt{\frac{\pi }{N}}$$

The corrected SI ranges from $-\frac{\gamma_{N}}{\left(1-\gamma_{N}\right)}$ to 1 where 1 indicates perfect synchronisation, and this correction is particularly important when sample size is low. Significance was assessed using the Rayleigh test, applied to the uncorrected mean resultant length.^36^ Only individuals with three or more recorded menstruation periods and a significant dominant multiday heart rate rhythm were included in the individual-level synchronisation analysis, consistent with the minimum heart rate data duration requirement.

#### Multinomial logistic regression

A multinomial logistic regression model was applied to the chronotype classifications to explore links between multiday chronotype (as the outcome) and specific demographic or behavioural factors. The following equation describes the multinomial logistic regression:

$$P\left(Y=k|X\right)=\frac{e^{\beta_{0k}+\beta_{k}^{T}X}}{1+\sum_{j=1}^{K-1}e^{{\beta_{0j}+\beta }_{j}^{T}X}}$$

where P(Y=k|X) is the probability of the outcome Y belongs to class k (i.e., weekly, monthly, multi-month chronotype) given the predictor variables X (sex, physical activity category, circadian chronotype and sleep duration category). The reference for class k is the weekly chronotype and the reference categories for predictor variables are female, very active, morning type, and longer sleep. $e^{{\beta_{0k}+\beta }_{k}^{T}X}$ represents the unnormalised class score or linear predictor for class k, found through the weighted sum of the predictors $\beta_{k}^{T}X$ and $\beta_{0k}$. The denominator adds up the total strength across all classes and normalises the class scores, to ensure probabilities across all outcome classes sum to one.

The model coefficients provide an indicator of the probability of belonging to a given outcome category in comparison to the reference categories: weekly multiday chronotype, female, very active, morning chronotype and longer sleep. Higher coefficient values reflect greater odds that a given demographic or behavioural category belongs to the outcome chronotype category, similar to effect size.

#### Assessing synchronicity

The influence of synchronicity of multiday cycles amongst individuals was considered. Each individual’s global wavelet periodogram was compared against other individuals (who were also study participants) in their self-reported social network. Individuals in the social network were grouped by interpersonal closeness, specifically ‘Close’ and ‘Not Close’ categories (as described in detail in Supplementary Appendix 5). Two metrics were employed to compare the similarity of periodograms between pairs–Peak Similarity and Wasserstein Distance. To assess whether there was a significant difference in Peak Similarity and Wasserstein Distance between ‘Close’ and ‘Not Close’ individuals, Generalised Estimating Equations were applied with a Gaussian family and exchangeable correlation structure. As individuals could contribute to multiple pairwise observations within the social network, GEE was used to account for the resulting within-individual correlation by clustering observations per individual.

### Role of funders

None of the funders had a role in study design, data collection, data analyses, interpretation, or writing of report.

## Results

The primary aim of the current study was to assess the prevalence of infradian rhythms in heart rate, with a focus on weekly to monthly cycles i.e., between 4 and 45 days (henceforth referred to as ‘multiday rhythms’). Heart rate data was recorded from 623 participants, from which 98 were excluded due to insufficient data (<3 months) for cycle detection, leaving 525 (262 female). The final dataset (50% female participants) remained representative of the original cohort in terms of sex (49% female participants). The mean recording duration across these individuals was 648.5 days (minimum: 93 days; maximum: 1391 days; standard deviation, SD = 444.1 days). The mean proportion of missing data was 12.1% (SD = 6.7%), with a mean missing segment duration of 3.2 days (SD = 3.7 days). Missing data did not substantially affect results, as similar cycles were observed when analyses were restricted to heart rate data with no more than 5 consecutive days missing (Supplementary Fig. S5). Further data characteristics are provided in Table 1 and Supplementary Table S1.

**Table 1:** Data characteristics.

	Number of Participants
Sex	
Male	317
Female	306

MEQ chronotypes	Summer 2015	Winter 2016
Definite evening	5	11
Moderate evening	71	132
Intermediate	342	379
Moderate morning	57	24
Definite morning	1	1
(No Response)	147	76

Summary of participant characteristics, including sex (female or male) and Morningness-Eveningness Questionnaire (MEQ) Chronotype. The MEQ is a 19-item self-report instrument assessing circadian disposition. Total scores (ranging from 16 to 86) classify chronotype from ‘Definite Evening’ to ‘Definite Morning’ (Definite Evening: 16–30; Moderately Evening: 31–41; Neither: 42–58; Moderately Morning: 59–69; Definite Morning: 70–86).^37^

### Multiday rhythmicity in human heart rate

**Fig. 2:**
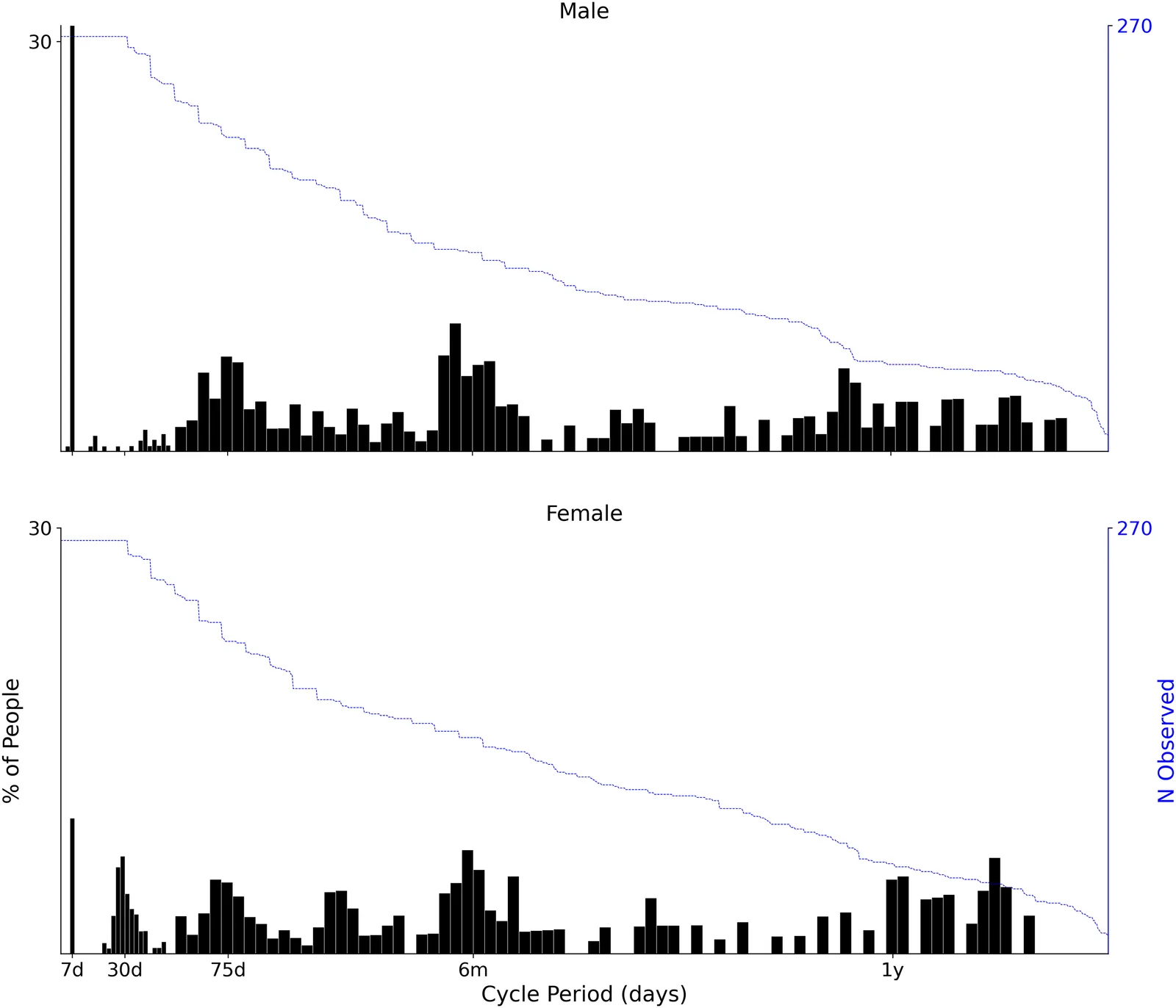
**Prevalence of all infradian heart rate rhythms.** Histograms showing the proportion of people (broken down by sex) with a significant detected rhythm at each cycle period (x-axis), with bar widths increasing from 2 days to 5 days at the 52-day mark. The y-axis on the right-hand side shows the sample size used at each cycle period, noting that the number of people with sufficient data duration decreases with increasing cycle lengths. The maximum period assessed for each individual was one-third of the total data duration, allowing a minimum of three cycles to be observed before significance was determined.

Overall, 70% (369/525) had at least one significant rhythm of ≥4 days and 36% (187/525) of people had two or more, with significant rhythms determined from peaks in individuals’ wavelet periodograms (see Fig. 1 and Supplementary Appendix 6). The distribution of all significant infradian rhythms showed clusters of individuals with rhythms at approximately 10-weeks, biannual, and annual periods, in addition to clusters associated with weekly and monthly periods (Fig. 2); this distribution was significantly non-uniform (KS statistic = 0.63, p < 0.001). Considering only the single most dominant peak per individual did not change the overall distribution of infradian rhythms (Supplementary Appendix 7).

Table 2 shows the number of people who had significant cycles at weekly (between 5 and 9 days), monthly (between 25 and 35 days), 10-week (between 8 and 12 weeks), biannual (between 5 and 7 months) and annual (between 11 and 13 months) periodicities. Interestingly, 48% of 234 participants observed had a significant biannual cycle in their daily heart rate, while 36% (36/99) had a significant annual cycle and 26% (98/383) had a significant 10-week cycle. The most common dominant cycle was biannual (found in 36% of 234 individuals), followed by annual (found in 22%, 22/99) and 10-week (found in 15%, 56/383) cycles. Weekly cycles were predominantly observed in males (77% of 107 observed weekly cycles), and monthly cycles were predominantly observed in females (97% of 61 observed monthly cycles). The mean intra-subject autocorrelation coefficient in the frequency domain over time was 0.68 (average SD = 0.21), demonstrating that these rhythms were relatively stable over time (Supplementary Appendix 8).

**Table 2:** Number of individuals with cycles at different multiday periodicities.

Cycle duration	Sample size	N strongest cycle	% Female	% Male	N significant cycle	% Female	% Male
Weekly 5–9 days	525	50 (9.5%)	28	72	107 (20.3%)	23.4	76.6
Monthly 25–35 days	505	38 (7.5%)	94.7	5.3	61 (12.1%)	96.7	3.3
10-week 8–12 weeks	383	56 (14.6%)	42.9	57.1	98 (25.6%)	42.9	57.1
Biannual 5–7 months	234	84 (35.9%)	51.2	48.8	113 (48.3%)	46.9	53.1
Annual 11–13 months	99	22 (22.2%)	36.4	63.6	36 (36.4%)	41.7	58.3

Table reporting the breakdowns by sex for individuals whose strongest cycle and significant cycle(s) fall within each cycle-duration category (i.e., 5–9 days, 25–35 days, 8–12 weeks, 5–7 months, 11–13 months). For each category, the overall sample size is provided, reflecting the reduction in sample size for longer periodicities due to variation in length of data recordings across participants.

**Fig. 3:**
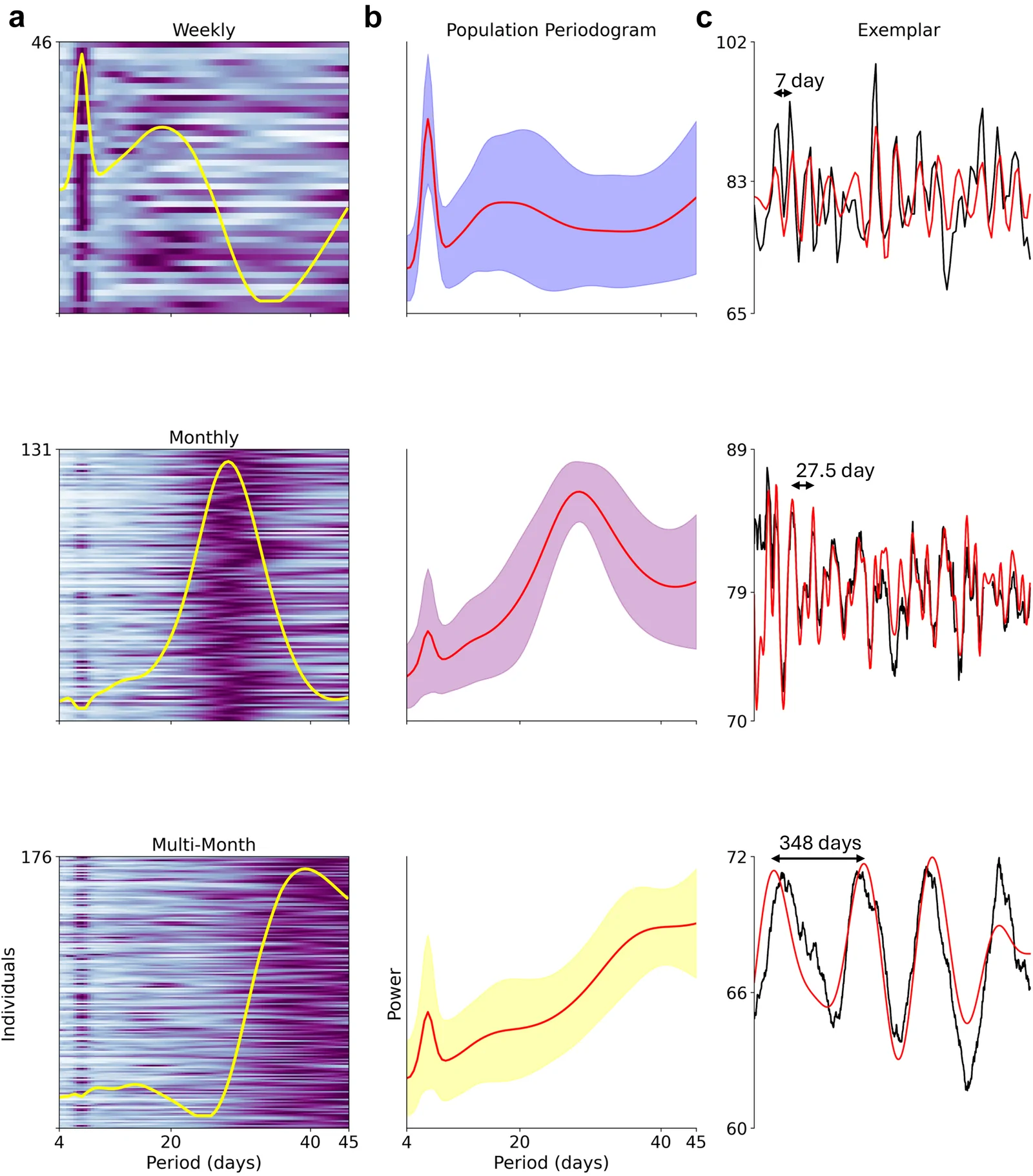
**Multiday chronotypes identified from heart rate cycles. a)** Heatmaps visually represent patterns of cyclic heart rate fluctuations within different chronotypes. Each row represents 1 individual periodogram derived from fluctuations in mean daily heart rate. Grouping of participants (n = 353) into three chronotypes is based on weights associated with components derived by nonnegative matrix factorisation (overlaid colour trace; see Methods); **b)** Mean of individuals’ periodograms (i.e., time-averaged wavelet power across each candidate period) for individuals belonging to the same chronotype. Shaded area is ±1 standard deviation of the power at each candidate period. Wavelet power reflects the strength (energy) of a given frequency/period component in the signal. Peaks indicate periods that contribute more strongly to the heart rate signal; **c)** Example of mean daily heart rate from one individual within each cluster, showing daily resting heart rate (in black) smoothed with a 2-, 7-, and 87-day moving average filter, respectively, and overlaid filtered cycle estimate (in red). Different scales are given for each example.

This study next explicitly considered multiday cycle periods from 4 to 45 days. Similarities in wavelet periodogram distributions across the cohort of individuals with at least 135 days of heart rate data and significant rhythms (n = 353) were identified using an unsupervised component decomposition (non-negative matrix factorisation; NMF). Common distributions, referred to as *multiday chronotypes*, were learnt from the data (Fig. 3), with three components chosen due to highest feature cross-correlation values (Supplementary Fig. S6). These components had peaks centred around 7-days (weekly, n = 46), 30-days (monthly, n = 131), and longer (multi-month, n = 176) periods.

Sex distribution differed significantly across chronotypes (Chi-squared test of independence, p < 0.0001). Weekly chronotypes were predominately observed in male participants (85%, 39/46), monthly chronotypes were predominantly observed in females (79%, 104/131), and multi-month chronotypes were predominantly observed in males (63%, 110/176).

### Environmental effects

**Fig. 4:**
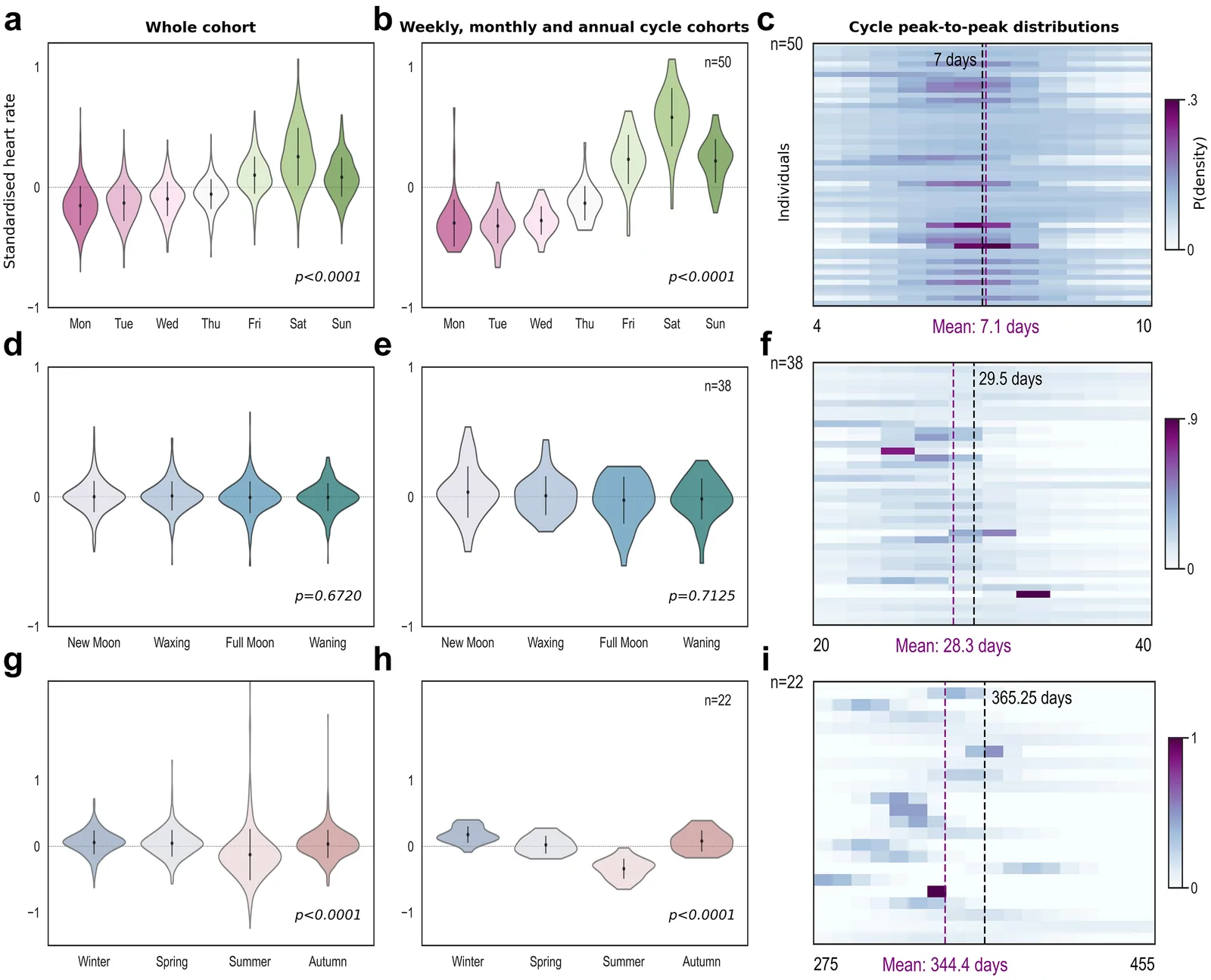
**Within-individual standardised heart rate across environmental factors (day-of-week, lunar and seasonal cycles).** Day-of-week (7 day) influence demonstrated in **a, b and c**, lunar (29.5 day) influence demonstrated in **d, e and f**, and seasonal (365.25 day) influence demonstrated in **g, h and i**. Heart rate data were first standardised within individuals and then means of the standardised heart rate were calculated per cycle phase (e.g., Winter, Spring, Summer and Autumn), for each individual. The first column (**a, d, g**) plots the distributions of individuals’ means of standardised resting heart rate values across phases of the environmental cycle, for the whole cohort (n = 525 per phase, per environmental factor). The second column (**b, e, h**) also plots the distributions of individuals’ means of standardised heart rate values across phases of the environmental cycle, but only for people whose strongest cycle was (**b**) weekly, defined as 5–9 days, (n = 50) (**e**) monthly, defined as 25–35 days, (n = 38) or (**h**) annual, defined as 11–13 months (n = 22). There was a significant (p < 0.0001, Kruskal–Wallis test) difference between at least one comparison of phases in the day-of-week and seasonal conditions, across the whole cohort and strongest cycle cohort. The third column (**c, f, i**) shows heatmaps of the distributions of peak-to-peak durations (days) for individuals with their strongest cycle being weekly, monthly or annual, respectively. Peak-to-peak durations were calculated using a peak detection algorithm on the filtered cycle, where the passband included frequencies within the full-width-half-maximum of the cycle peak from the individual’s original periodogram. Each row in the heatmap represents one individual. Vertical purple dashed lines represent mean peak-to-peak durations across the cohort, and black dashed lines represent the true environmental cycle duration (7 days, 29.5 days and 365.25 days, respectively). The colour intensity represents the probability density (relative likelihood) of observing each peak-to-peak duration in days for each subject. Wider distributions indicate greater variability and reflect non-stationary cycle dynamics, while narrower distributions correspond to more stable, stationary cycles.

Environmental drivers were considered, including day-of-week, lunar phase and seasons. Both the day-of-week and the season were significantly (p < 0.0001, Kruskal–Wallis) associated with variation in heart rate across the cohort, as displayed in Fig. 4. There appeared to be a “weekend effect”, with higher heart rate values across the cohort visually evident on Friday and Saturday (Fig. 4a). There were also seasonal effects on heart rate across the cohort, with lower average heart rate visually apparent during the summer in comparison to winter (Fig. 4g). Conversely, the lunar cycle did not appear to significantly affect resting heart rate on a whole cohort level nor in individuals with dominant monthly heart rate rhythms.

Day-of-week and seasonal effects observed across the cohort appeared stronger in individuals with significant dominant cycle in the weekly (5–9 day) and annual (11–13 month) range (Fig. 4b and h), suggesting that these cohort-wide weekly and annual trends are partially driven by a subset of individuals. However, even in these individuals, the peak-to-peak durations of the significant dominant weekly or annual cycle was not closely aligned with the environmental driver (day-of-week or season), with few exhibiting tight distributions around the fixed-period environmental cycles of 7 days and 365.25 days (Fig. 4c and i).

Notably, when all three environmental factors (day-of-week, lunar phase and season) were simultaneously regressed out of individuals’ heart rate signals using the individualised GAM models (Supplementary Fig. S13), significant cycles remained for 64% (n = 338/525). However, there was a marked reduction in the prevalence of the weekly (from 20% of 525 individuals to 6%), monthly (from 12% of 505 to 4%), biannual (from 48% of 234 to 28%) and annual (from 36% of 99 to 28%) cycles (Supplementary Table S2), while other cycles remained at a similar prevalence or even increased in prevalence (e.g., 10-week rhythm increased from 26% of 383 to 29%, Supplementary Fig. S13). Nevertheless, significant rhythms from about-weekly to about-monthly (4–45 d range) were still identified in 29% (n = 137/472) following environmental regression, with periods that were not aligned to environmental zeitgebers. For instance, the 31 people (6% of 525) with significant about-weekly rhythms (Supplementary Table S2) showed mean peak-to-peak distance of 5.8 d (desynchronised from the 7 d week).

We also observed significant day-of-week, lunar and seasonal changes in sleep duration and physical activity minutes across the cohort and in individuals with significant weekly, monthly and annual rhythms (Supplementary Appendix 10). However, daily resting heart rate was not strongly correlated to daily physical activity and sleep duration (Supplementary Figs. S1 and S2).

### Hormonal effects

**Fig. 5:**
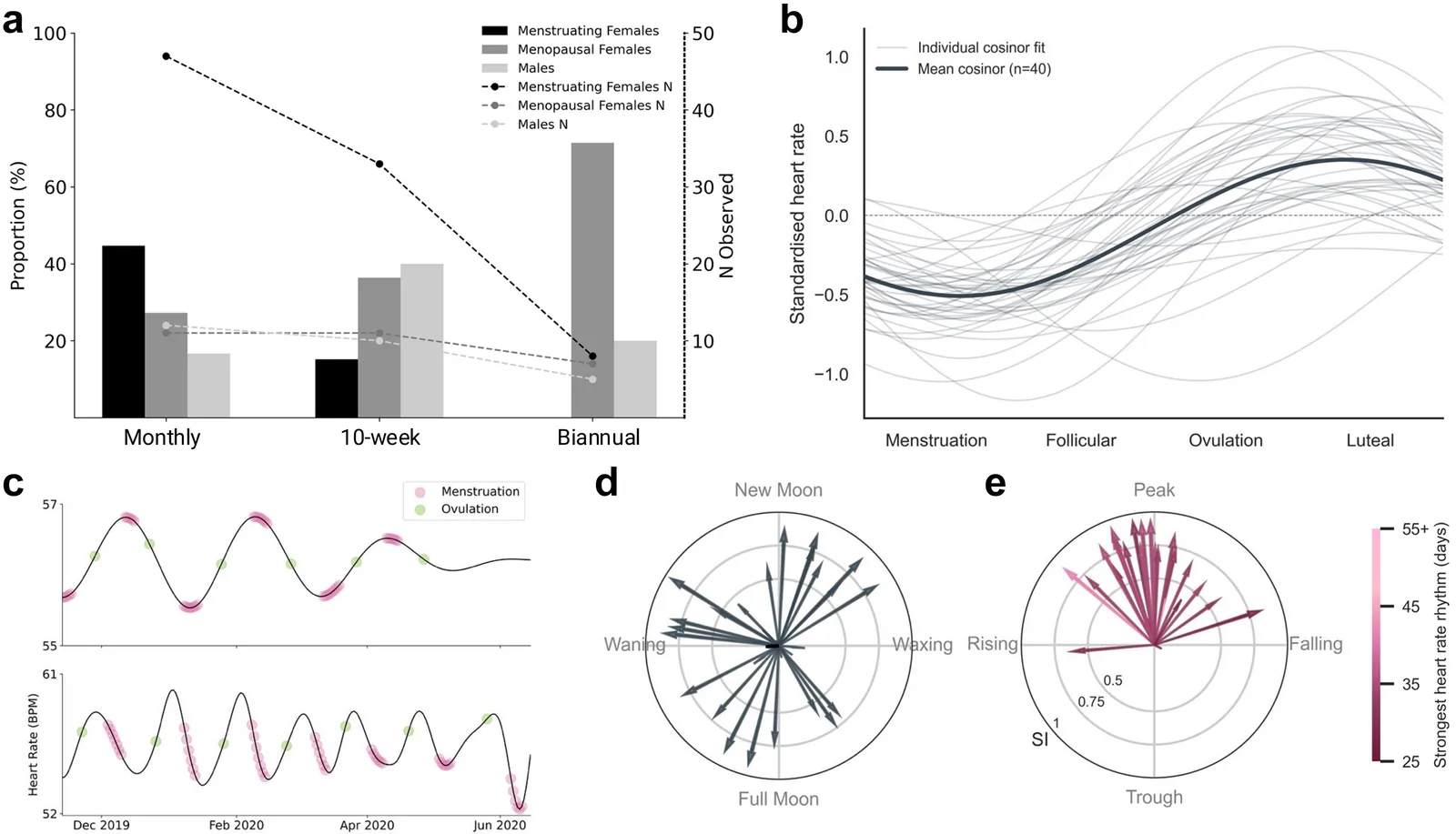
**Distribution of multiday heart rate rhythms and association to menstruation within a cohort of 70 adults (47 menstruating females, 11 menopausal females and 12 males). a.** Prevalence (%) of monthly (25–35 days), 10-week (8–12 weeks) and biannual (5–7 months) cycles in menstruating females (black), menopausal females (dark grey) and males (light grey). The y-axis on the right-hand side shows the sample size used at each cycle period, noting that the number of people with sufficient data duration decreases with increasing cycle lengths. **b.** Cosinor fit of standardised daily resting heart rate values across menstrual cycle phases (i.e., menstruation, follicular, ovulation, luteal) in menstruating females (light grey lines) with a significant non-uniform distribution (n = 40/45, 89%). The mean cosinor fit across the significant cohort (dark grey line) shows resting heart rate peaks during the luteal phase. **c.** Two individual menstruating female examples of a heart rate rhythm, i.e., filtered cycle estimate of the individual’s strongest cycle (black), with menstruation and ovulation days dotted in pink and green, respectively. **d.** Individuals’ Synchronisation Index (SI, length of arrows) of menses onset with respect to the lunar cycle (n = 43 with ≥3 recorded menstrual cycles). **e.** Individuals’ SI of menses onset with respect to their strongest heart rate cycle in days (n = 35 with ≥3 recorded menstrual cycles and a significant heart rate cycle). Darker colours representing shorter rhythms and lighter colours representing longer rhythms. Monthly (25–35 days) rhythms were the most dominant heart rate rhythms in 60% (n = 21/35) of these females.

The relationship between heart rate rhythms and menstruation was also investigated (Fig. 5) in a separate cohort (Soochow University, China), which captured longitudinal heart rate data alongside menstrual periods or ovulation in the menstruating females. Seventy individuals in this cohort had sufficient heart rate data recorded for analysis (47 menstruating females, 11 menopausal females and 12 males) and 76% (n = 53/70) had a significant multiday heart rate rhythm.

Significant monthly rhythms were most prevalent in menstruating females (observed in n = 24/47), although were still observed in menopausal females (n = 3/11) and males (n = 2/12); 10-week cycles were most common in males (n = 4/10), followed by menopausal females (n = 4/11) and menstruating females (n = 5/33); and biannual (5–7 month) rhythms were most common in menopausal females (n = 5/7) followed by males (n = 1/5), with no biannual rhythms observed in menstruating females (Fig. 5a). No weekly (5–9 day) rhythms were observed in this cohort, and there was insufficient data to observe yearly/seasonal rhythms.

The menstrual cycle was significantly (p < 0.05, F-test) associated with changes in resting heart rate in most menstruating females (n = 40/45, after removing n = 2/47 with outlier cycle periods), with cohort-average resting heart rate peaking during the luteal phase and at its lowest between menstrual and follicular phases (Fig. 5b). This association appeared to be linked to the synchronisation between menses onset and the dominant heart rate rhythm (p < 0.05 in n = 17/35 females with ≥3 recorded menstrual cycles and a significant heart rate cycle) (Fig. 5e), with a median corrected Synchronisation Index (SI) of 0.65 (Range: −0.43–0.94) observed across the cohort. Menses onset usually occurred around the peak of the heart rate rhythm. Synchronisation between menstruation and heart rate rhythm phase was stronger in individuals whose dominant heart rate rhythm was in the monthly (25–35 day) range (Median SI = 0.74; p < 0.05 using Rayleigh test in n = 15/21) vs. non-monthly (Median SI = −0.19; p < 0.05 in n = 2/14). These results suggest a coupling of multiday resting heart rate rhythms to circulating reproductive hormones in many menstruating females.

In contrast, the median SI between menses onset and the lunar cycle was 0.44 (p < 0.05 in n = 13/43 with ≥3 recorded menstrual cycles). Phases were dispersed across the lunar cycle rather than clustered around any particular phase (Fig. 5d), which does not support environmental entrainment of menstruation onset by the lunar cycle.

### Demographic and behavioural effects

**Fig. 6:**
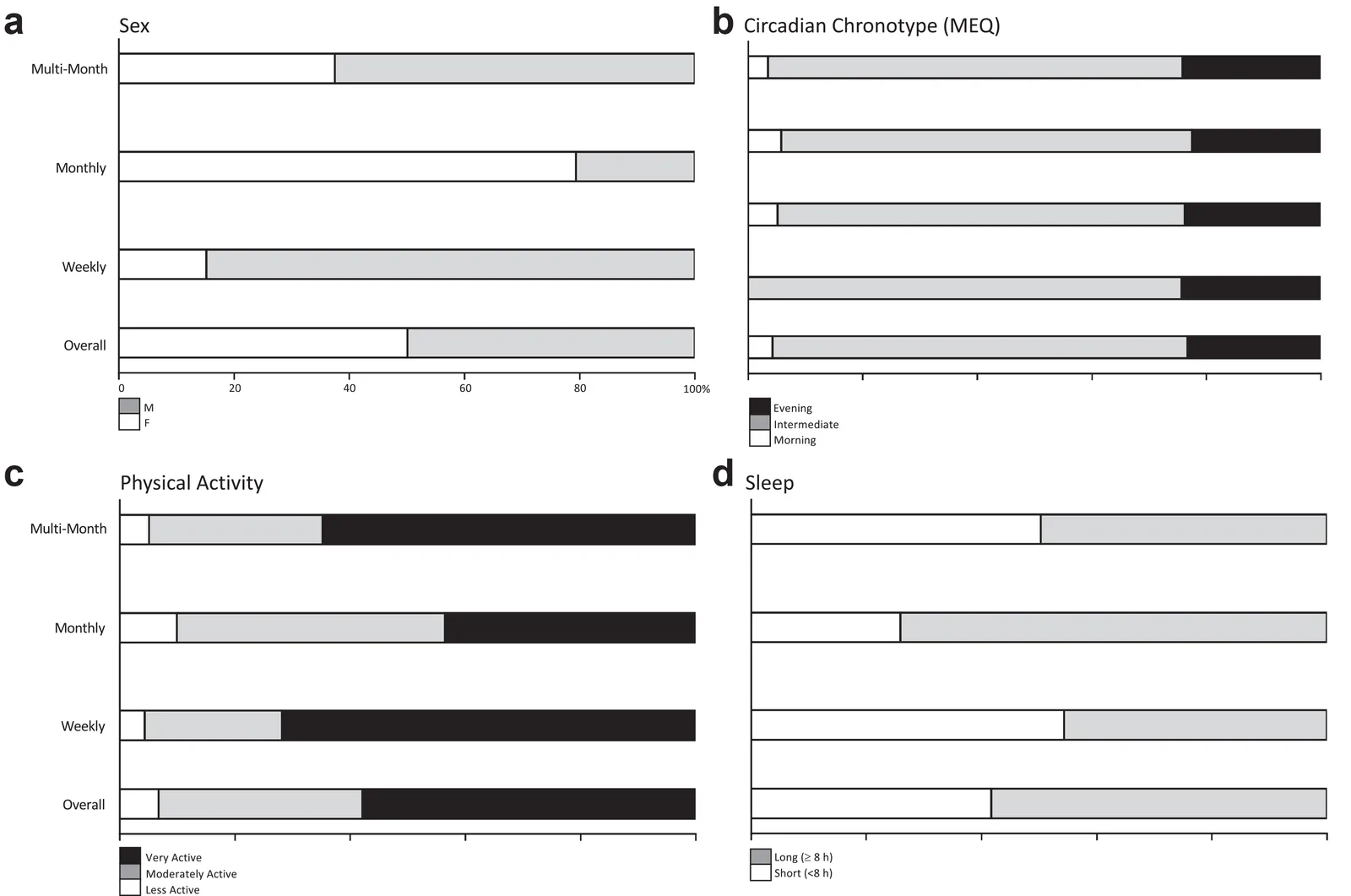
**Distribution of multiday chronotypes according to demographic and behavioural factors.** Subpanels show the proportion of people (n = 353) with each multiday chronotype based on **a) Sex**, **b) Circadian chronotype**, i.e., the preferred sleep timing assessed from self-reported MEQ survey instrument (here ‘definite’ and ‘moderate’ morning/evening types were combined). **c) Weekly activity level** based on the mean active minutes per week (measured from the wearable device) and defining less active as <150 mins per week, moderately active as 150–300 mins per week and ‘very active’ as >300 min per week. The distribution of activity minutes is given in Supplementary Fig. S16. **d) Sleep duration** recorded from wearable devices, with ‘short sleep’ defined as <8 h, ‘long sleep’ defined as ≥8 h. Associations between demographic and behavioural factors with multiday chronotype were analysed using multinomial logistic regression (Supplementary Table S3).

Fig. 6 shows the demographic and behavioural characteristics of each multiday chronotype (weekly, monthly, multi-month), according to individuals’ sex, circadian chronotype preference (morning/intermediate/evening), average activity level (less, moderately and very active) and average sleep duration (short, <8 h, and long, ≥8 h). Activity levels and sleep duration were categorised according to average American guidelines (Supplementary Appendix 11).

Multinomial logistic regression was used to model significant predictor variables to investigate the associations between these factors and multiday chronotype. As seen in Fig. 6 and confirmed by the model (Supplementary Table S3), sex was the only significant predictor of multiday chronotype, where males had significantly lower odds of a monthly chronotype relative to weekly (OR = 0.06, 95% CI [0.02, 0.16], p < 0.0001) and lower odds of a multi-month chronotype relative to weekly (OR = 0.30, 95% CI [0.12, 0.74], p = 0.009), compared to females. Physical activity, circadian chronotype and sleep duration were not significant predictor variables of multiday chronotype, although both physical activity and sleep correlated with daily heart rate at individual level (Supplementary Appendix 1).

Finally, the phenomenon of physiological synchrony was explored by comparing the wavelet periodogram distributions and peaks between pairs of individuals with close (i.e., roommates, siblings or friends) and socially distant (i.e., strangers) relationships (Supplementary Fig. S7). Close pairs demonstrated significantly higher similarity in their wavelet periodogram peaks compared to socially distant pairs (p = 0.01, GEE), however the overall shape of the periodogram distributions, as measured by Wasserstein Distance, did not differ significantly between groups (p = 0.06, GEE), suggesting that while close individuals tend to share similar dominant heart rate cycles, their broader spectral profiles remain comparable to those of socially distant pairs.

## Discussion

Infradian, or multiday, rhythms of resting heart rate were found in over two-thirds of young (college-aged) adults, commonly with weekly, monthly or longer (e.g., 6-and 12-month) periods. Cycles were not necessarily linked to environmental cues, such as the day-of-week, lunar phase or seasonal transitions. Despite evidence of weekday effects, weekly cycles were not the strongest cycle for most people and persisted when environmental effects were regressed out of the model. Indeed, the most common rhythms were biannual (6-month) and 10-week cycles, which lack any clear environmental or behavioural explanation. Sex was significantly linked to cycle period, with males being more likely to have weekly and multi-month rhythms, and females more likely to have monthly rhythms. Given the prevalence of multiday heart rate rhythms amongst healthy young people, it is vital to understand their underlying mechanisms and possible effects on both healthy function and disease states. This study represents a fundamental step towards elucidating widespread, individual-specific infradian rhythms affecting human physiology.

The widespread detection of multiday rhythms and their heterogenous timescales support the existence of intrinsic mechanisms alongside known environmental modulation. While evidence of multiday rhythms of heart rate in the general population remains limited, endogenous cycles of cardiac activity have previously been identified in individuals with pacemakers,^13^ epilepsy cohorts,^12^^,^^29^ and premature infants.^3^ The current work confirms the findings of these earlier studies and further extends the understanding of multiday autonomic rhythms beyond narrow cohorts and disease-specific effects to fundamental physiology. Robust annual and biannual rhythms in heart rate were observed in nearly half of the cohort, consistent with previous observations in clinical populations.^13^ While seasonal factors significantly affected heart rate—with peaks in winter and troughs in summer—this modulation likely reflects a complex interplay of environmental photoperiods, temperature, and seasonal shifts in physical activity. Such circannual variation is well-documented in metabolic^37^ and hormonal secretion.^9^^,^^38^^,^^39^ Nevertheless, several lines of evidence argue against a purely exogenous driver for these cycles. First, the peak-to-peak durations of annual heart rate rhythms were not precisely locked to the 365-day solar year, suggesting that the environmental cycle may entrain, but does not entirely account for these oscillations. Second, the prevalence of biannual (48%, 113/234) and 10-week (26%, 98/383) rhythms cannot be explained by known geophysical or social influences. While biannual rhythms may represent a harmonic of the annual cycle, the 10-week periodicity lacks any clear intrinsic or extrinsic precedent, though it may emulate the quarterly (3-month) cycles observed in patients with cardiac pacemakers.^13^ Thus, the evidence in this study points toward a conserved, long-term regulatory mechanism of the autonomic nervous system. Further investigation is required to elucidate the underlying drivers of these biannual and 10-week oscillations, which appear to be fundamental, yet under-recognised, features of human physiology.

Another central finding of this work was the presence of monthly rhythms across the entire cohort, including in males and menopausal females (albeit to a lesser extent than menstruating females). Previous work has demonstrated that menstruating females exhibit higher resting heart rates during the luteal phase,^40^ a finding that was corroborated in this work. Advancing on this, we showed that menstruation times were strongly synchronised to heart rate rhythm phase in most menstruating females and had a consistent phase of alignment across individuals compared to lunar synchronisation. While previous research has proposed evidence of an endogenous circa-monthly clock governing menstrual periods^5^ these results suggest coordination between the reproductive system and the cardiovascular system on a circa-monthly timescale, without evidence of the moon as an environmental zeitgeber. Although, desynchronisation from the synodic month may derive from modern exposure to artificial light.^5^ Furthermore, the existence of monthly rhythms in males and menopausal females across this cohort and others,^12^^,^^24^^,^^26^ as well as the prevalence of rhythms at other periodicities, indicates that the potential for endogenous infradian clock mechanisms is not restricted to female reproductive hormones.

Nevertheless, the striking differences in infradian rhythm periods and multiday chronotypes between males, menstruating females and menopausal females, suggests that the architecture of multiday coupling changes across sex and life stages. This is corroborated in some animal models, where infradian rhythms differ by age and sex,^41^ and across the oestrus cycle.^42^ In other human studies, monthly rhythms were rare in a cohort of 336 individuals with pacemakers,^13^ which likely reflects an older cohort; in fact, annual rhythms were by far the most common, found in two-thirds of the cohort, followed by quarterly (12%) and weekly (12%) rhythms. In the present study, weekly rhythms were observed in 20% of 525 individuals, predominately males, which may be partially explained by shorter reproductive hormone rhythms documented in males.^4^ On the other hand, day-of-week effects were significantly associated with weekly rhythms in this cohort, which could be related to weekly behavioural changes, such as higher alcohol consumption or deviations from normal bed- and wake-times over the weekend, both known to transiently elevate resting heart rate.^30^ Notably, correcting for day-of-week substantially reduced the prevalence of precise 7-day rhythms, albeit not other about-weekly rhythms. This aligns with previous research in people with epilepsy, where about-weekly rhythms in seizure timing,^16^ brain excitability^26^ and heart rate^12^ are frequent but rarely precisely 7 days.

There are several proposed theories of the origin and biological processes responsible for controlling and synchronising biological rhythms in the body, although the mechanisms of multiday rhythms remain unknown. The suprachiasmatic nucleus (SCN) in the hypothalamus is well-recognised as the core circadian clock, controlling daily physiological processes and enabling alignment to environmental conditions.^43^ Peripheral clocks are circadian oscillators located in tissue and organs; while they were historically thought to be synchronised by the SCN, emerging evidence suggests that they are not entirely reliant on the SCN^43^^,^^44^ and have the ability to synchronise with other organs and tissues through intercellular coupling and paracrine signalling.^43^ This ability of peripheral clocks to oscillate independently from the SCN forms the basis for proposed hypotheses as to the origin of infradian rhythms; more specifically, since peripheral clocks are not necessarily entrained to environmental cues (i.e., the light–dark cycle of the solar day) they could oscillate with slower frequencies and follow internal cues.^44^ Similarly, dopaminergic neural oscillators capable of infradian rhythm generation appear independent from core clock circuitry.^45^^,^^46^ Thus, a possible mechanism of multiday rhythms is that physiological systems consisting of coupled oscillators drive longer, emergent rhythmic dynamics, within a broad range of timescales,^47^ consistent with the observed heterogeneity of heart rate cycles.

Beyond individual biological pacemakers, our findings suggest that infradian rhythms are associated with social and environmental proximity. Individuals in close relationships (i.e., roommates, siblings and friends) exhibited significantly higher similarity in their multiday chronotypes and rhythm periodicities compared to strangers, suggesting a form of interpersonal physiological synchrony—a phenomenon previously documented in short-term contexts such as parent-child interactions,^48^ friendships,^49^ and those performing group tasks^32^^,^^50^^,^^51^ and sharing experiences.^49^ Our results extend these observations to a much longer timescale, suggesting that shared social zeitgebers may entrain the autonomic nervous system over weeks and months. While anecdotal evidence for long-term synchronisation (most notably the ‘McClintock effect’ in menstrual cycles^52^) remains a subject of scientific debate, our data supports the existence of shared infradian rhythmicity among socially connected individuals and, combined with the present findings on menstrual cycle/heart rate rhythm synchronicity, may even partially explain the McClintock effect. While sibling sample sizes were insufficient to suggest a genetic driver in this work, future studies are warranted to decouple the relative contributions of genetic architecture vs. shared environmental entrainment in governing these long-term biological clocks.

The characterisation of multiday rhythms has potential to be used for individualised, chronotherapeutic disease management. This approach is validated in the management of chronic neurologic conditions like epilepsy, where monitoring multiday seizure cycles can improve health outcomes and quality-of-life.^24^^,^^53^^,^^54^ The striking similarity in prevalence between the heart rate rhythms observed here in healthy adults and in clinical cohorts^13^^,^^15^^,^^24^ suggests that multiday periodicity is a fundamental property of the human nervous system. While the biological impact of multiday rhythms is unexplored, recent research suggests links to salivary cortisol^55^ and brain excitability.^12^^,^^56^ Thus, monitoring these rhythms could provide a window into periods of heightened susceptibility to stress, which may be relevant for the management of psychiatric disorders that fluctuate over extended timescales, such as seasonal affective disorder and bipolar disorder. Moreover, multiday heart rate rhythms have been found in cardiovascular disease, alongside exacerbations in disease symptoms at specific circadian,^57^ seasonal^19^ and even weekday^58^ phases. While the cyclic risk profiles of cardiac disease have mostly been studied at the population-level, tracking individual-level cycles could be an additional time-varying biomarker of the risk of cardiac arrhythmias.

In women’s health, routine tracking of infradian biology has already seen widespread adoption, with tracking of hormonal and menstrual cycles used for fertility and lifestyle planning.^59^ However, there are limitations in accuracy and consideration of variability between individuals, especially when the cycle does not conform to a 28-day timeframe.^59^ The findings presented here suggest that heart rate rhythms may be utilised to improve hormonal and menstrual cycle tracking, particularly during periods of heightened stress,^60^ which is known to affect menstrual period regularity.^60^ Thus, by leveraging the acquisition of heart rate data via wearable sensors, heart rate rhythms have potential to provide a longitudinal proxy for reproductive and stress hormones, although further study is undoubtedly required. Nonetheless, this shift toward continuous biometric monitoring reduces reliance on discrete manual logging, offering a scalable approach to characterise individual variability and non-stationarity in reproductive physiology across diverse populations.

This study has several limitations. First, this study primarily analysed the NetHealth dataset, which comprised longitudinal heart rate recordings from a relatively homogeneous cohort of young adults aged 17–23 years. Although participants varied in demographic background and had diverse geographic origins across the United States, they shared broadly similar lifestyles as students at a single university and resided predominantly in the same location during the four-year study period, which may limit generalisability. Future work should test the robustness of these multiday rhythms in larger, more diverse cohorts spanning wider age ranges, geographic contexts and socioeconomic backgrounds, and should incorporate family- or twin-based designs to quantify potential genetic contributions. Additionally, it would be valuable to consider the role of other health metrics (e.g., sleep stage, body temperature and blood oxygen) on infradian rhythms, many of which can be accurately captured using modern wearable devices. Diet, caffeine, drug, and alcohol consumption should also be captured in future studies to understand the acute effects of these behaviours on heart rate.

Second, while the NetHealth study provided longitudinal datasets spanning up to four years, detecting longer infradian rhythms (e.g., annual, multi-year, or decadal rhythms) would require substantially longer continuous recordings. The present analysis and findings were therefore limited to rhythms of up to a little over a year, particularly given that the analysis of cycle periods were restricted to one-third of an individual’s recording length and the substantial reduction in wearable coverage across participants over time. Consequently, longer timescales were not considered in this study, and future work should leverage not only larger, more diverse cohorts but also extended datasets. Considering commercially available wearable devices emerged in the late 2000s and early 2010s, it is now feasible that a growing number of individuals could have data spanning close to a decade, enabling investigation of longer physiological rhythms.

Third, statistical significance does not guarantee the existence of a true biological rhythm. Rather, the statistical significance threshold utilised in the wavelet analysis to identify underlying cyclicality in the data indicates that any cyclicality surpassing this threshold is highly unlikely to have occurred due to random chance. Given the vast number of individuals and periodicities tested in this work, some false discoveries are unavoidable; thus, the significant rhythms reported should be interpreted as candidates warranting further investigation rather than definitive evidence of an underlying biological process. Nevertheless, the reproducible clustering of cycles at similar periodicities across individuals lends support to these findings reflecting an underlying physiological mechanism.

Fourth, as light exposure was not directly measured in this study, disentangling light-driven influences on infradian rhythms was not possible. Natural photoperiod varies seasonally and is a plausible contributor to the seasonal effects on heart rate observed here, particularly given its possible influence on sleep and exercise. Additionally, exposure to artificial light sources, including screens and indoor lighting, may vary across the week, thus modulating autonomic tone via melatonin suppression, potentially contributing to the day-of-week effects observed. Lunar light, which varies across the synodic cycle, has also been proposed as a potential modulator of melatonin and sleep,^61^ and may partially explain the weak but significant lunar effects observed in some individuals. Future studies incorporating continuous light monitoring alongside wearable heart rate would help to clarify the role of both natural and artificial light in driving infradian heart rate variation.

Finally, the menstrual cycle data from the Soochow dataset were self-reported, introducing potential recall bias in the timing of menstruation onset and selection bias toward more consistent trackers. Future work using passively sensed menstrual indicators, such as continuous hormone monitoring or skin temperature, would help to validate these findings without reliance on self-report. Further, analysis of the Soochow dataset served as a preliminary investigation of the relationship between infradian heart rate rhythms and the menstrual cycle. In particular, menstrual cycles are non-stationary and unique to individuals; thus, methods that assume stationarity and sinusoidal characteristics in menstrual cycles (such as the cosinor method used in this work) may not accurately capture the physiological association between menstruation and resting heart rate, particularly phase inference on unstable cycles. Future work should expand upon these findings in larger cohorts with waveform-agnostic methods to validate the observed associations between the menstrual cycle and infradian heart rate rhythms.

This study characterised infradian (multiday) heart rate rhythms in a general population, demonstrating that human cardiac physiology has a complex, multi-layered temporal architecture. The findings revealed that two-thirds of individuals exhibit robust multiday cycles, often independent of exogenous environmental influences. By identifying distinct multiday chronotypes associated with sex, sleep, and social proximity, we establish these oscillations as fundamental features of individual autonomic regulation. The integration of longer rhythms into wearable health platforms offers an increasingly relevant framework for women’s health tracking, monitoring and managing fluctuating neurological and psychiatric conditions, and potentially for cardiovascular health. Ultimately, these results reveal an under-recognised dimension of human physiology, reinforcing the need to investigate these rhythms over larger, more diverse cohorts and in the context of disease and personalised medicine.

## Contributors

R.I.D. contributed to data curation, formal analysis, investigation, methodology, software, visualisation, writing—original draft, and writing—review & editing.

R.E.S. contributed to conceptualisation, data curation, formal analysis, investigation, methodology, resources, software, supervision, validation, visualisation, writing—original draft, and writing—review & editing.

P.J.K. contributed to conceptualisation, funding acquisition, investigation, methodology, project administration, resources, supervision, validation, visualisation, writing—original draft, and writing—review & editing.

J.N.F. contributed to conceptualisation, investigation, methodology, supervision, validation, visualisation, writing—original draft, and writing—review & editing.

S.P. contributed to methodology, validation, and writing—review & editing.

E.P. contributed to methodology, and writing—review & editing.

R.I.D. & R.E.S. accessed and verified the underlying data.

All authors read and approved the final version of the manuscript.

## Data sharing statement

The datasets analysed during the current study are available in the University of Notre Dame’s (USA) NetHealth repository, https://sites.nd.edu/nethealth/ (heart rate for main cohort) and Mendeley, https://doi.org/10.17632/v58stpfcnm.1 (heart rate with menstrual cycles timing). The code supporting the analysis and findings of this study is available from the corresponding author upon request.

## Declaration of interests

Philippa J Karoly reports financial support from a National Health and Medical Research Council grant (2033596) and Australian Research Council Discovery Project grant (DP240102899). Philippa J Karoly reports support for attending meetings and travel from the Gordon Research Conference. Rachel E Stirling reports financial support from The University of Melbourne McKenzie Postdoctoral Fellowships Scheme. Rochelle De Silva reports financial support from an Australian Government Research Training Program Scholarship (doi.org/10.82133/C42F-K220). Elizabeth Paratz reports financial support from the Pfizer Medical Education Grant and has received speaking, lecturing, and consulting fees from Bristol Myers Squibb and Pfizer. Elizabeth Paratz reports consulting fees from DKSH Healthcare and CSL Sequirus.

Other authors declare that they have no known competing financial interests or personal relationships that could have appeared to influence the work reported in this paper.
