# Supplementary material for: Infradian rhythms of human heart rate reveal distinct multiday cycle periods in healthy adults
Source: eBioMedicine. 2026 Aug 22;131:106444. doi: 10.1016/j.ebiom.2026.106444 (PMC13524855; doi:10.1016/j.ebiom.2026.106444)
Supplement: Supplementary file 1 [file mmc1.docx]

# Supplementary Material

**Supplementary Table 1: Participant demographics and characteristics**

|  | | | | Number of Participants | | | | |
| --- | --- | --- | --- | --- | --- | --- | --- | --- |
| U.S. Region of Origin | | | |  | | | | |
| New England  *(i.e., Connecticut, Maine, Massachusetts, New Hampshire, Rhode Island, Vermont)* | | | | 24 | | | | |
| Middle Atlantic  *(i.e., New Jersey, New York, Pennsylvania)* | | | | 81 | | | | |
| East North Central  *(i.e., Illinois, Indiana, Michigan, Ohio, Wisconsin)* | | | | 222 | | | | |
| West North Central  *(i.e., Iowa, Kansas, Minnesota, Missouri, Nebraska, South Dakota, North Dakota)* | | | | 54 | | | | |
| South Atlantic  *(i.e., Delaware, Florida, Georgia, Maryland, South Carolina, North Carolina, Virginia, West Virginia, Washington D.C.)* | | | | 71 | | | | |
| East South Central  *(i.e., Alabama, Kentucky, Mississippi, Tennessee)* | | | | 15 | | | | |
| West South Central  *(i.e., Arkansas, Louisiana, Oklahoma, Texas)* | | | | 34 | | | | |
| Mountain  *(i.e., Arizona, Colorado, Idaho, Montana, Nevada, New Mexico, Utah, Wyoming)* | | | | 17 | | | | |
| Pacific  *(i.e., Alaska, California, Hawaii, Oregon, Washington)* | | | | 65 | | | | |
| Other | | | | 40 | | | | |
| Self-Reported Exercise Frequency Summer 2015 |  | | | | |  | | |
| 3 or more times a week | 305 | | | | | | | |
| 1-2 times a week | 124 | | | | | | | |
| 1-2 times a month | 30 | | | | | | | |
| Less than 1-2 times a month | 13 | | | | | | | |
| Not at all | 4 | | | | | | | |
| (No Response) | 147 | | | | | | | |
| Self-Reported Physical Activity |  | | | | | | | |
|  | **Summer 2016** | **Fall 2016** | **Spring 2017** | | **Fall 2017** | | **Spring** **2018** | **Spring 2018** |
| Very Inactive | 33 | 43 | 44 | | 46 | | 29 | 17 |
| Inactive | 78 | 78 | 84 | | 62 | | 51 | 25 |
| Moderately Active | 47 | 45 | 48 | | 47 | | 40 | 23 |
| Active | 120 | 114 | 94 | | 93 | | 76 | 48 |
| Very Active | 123 | 90 | 91 | | 64 | | 67 | 73 |
| (No Response) | 222 | 253 | 262 | | 311 | | 360 | 437 |
| PSQI Chronotypes | | | | | | | | |
|  | **Fall 2015** | **Spring 2016** | **Summer 2016** | | **Fall 2016** | | **Spring**  **2017** | **Fall**  **2017** |
| Good Sleep Quality | 122 | 76 | 123 | | 40 | | 48 | - |
| Poor Sleep Quality | 329 | 452 | 275 | | 306 | | 291 | - |
| Severe Sleep Difficulties | 13 | 17 | 2 | | 20 | | 17 | - |
| (No Response) | 159 | 78 | 223 | | 257 | | 267 | - |

### Appendix 1: Effects of physical activity and sleep

To account for the well-known relationship between heart rate and physical exertion, the effects of physical activity were investigated. We assessed the linear correlation between daily heart rate (considering either the daily mean, or 30^th^ percentile of heart rate) and physical activity for each participant individually. The number of active minutes per day was taken as the sum of moderate and high intensity activity, defined by Fitbit as ‘fairly active’ (3-6 METs) and ‘very active’ (>6METs), respectively. Days when the number of active minutes was zero were excluded from the estimation. In comparison to daily resting heart rate (Supplementary Figure 1a) (n = 237/525 with a significant correlation), individuals displayed a stronger linear coupling between activity and daily mean heart rate (Supplementary Figure 1b) (n=507/525 with a significant correlation).

**Supplementary Figure 1: Individual correlation between physical activity and both daily resting HR and daily mean HR.** R-values (x-axis) reflect the Pearson’s correlation between either daily resting HR or daily mean HR and minutes of physical activity per day. (a) Resting HR was derived as the average of the lowest 30^th^ percentile of HR values across the day. (b) Mean HR was derived as the average of HR values across the day.

The relationship between heart rate and daily sleep duration was also explored. Similar to the physical activity and heart rate analysis, linear correlations between daily heart rate (considering either the daily mean, or 30^th^ percentile of heart rate) and daily sleep duration were assessed for each participant individually. Daily sleep duration was calculated using Fitbit sleep data, specifically the recorded ‘mins asleep’ metric. Naps (i.e., sleep durations less than 4 hours) were excluded from the analysis. To account for wakefulness during the middle of sleep, sleep periods less than 4 hours were included only when the recorded ‘time to bed’ (i.e., sleep onset) and ‘time out of bed’ (i.e., wake) occurred within 1 hour of the expected sleep interval. In comparison to daily resting heart rate (Supplementary Figure 2a) (n = 296/525) with a significant correlation), individuals displayed a slightly stronger linear coupling between sleep and daily mean heart rate (Supplementary Figure 2b) (n=319/525) with a significant correlation)

**Supplementary Figure 2: Individual correlation between daily sleep duration and both daily resting HR and daily mean HR.** R-values (x-axis) reflect the Pearson’s correlation between either daily resting HR or daily mean HR and minutes of physical activity per day. (a) Resting HR was derived as the average of the lowest 30^th^ percentile of HR values across the day. (b) Mean HR was derived as the average of HR values across the day.

### Appendix 2: Study Flow Diagrams

Study flow (or CONSORT style) diagrams for both the NetHealth Study and the Soochow Study (Supplementary Figures 3 and 4 respectively) display a breakdown of total participants into those included and excluded from further analysis. Inclusion criteria for analysis requires participants to have at least 3 months of recorded heart rate data and 80% data completeness (NetHealth: n=525;262F and Soochow: n=70; 47MF, 11PMF, 12M). Those who did not meet this requirement (NetHealth: n=98;44F and Soochow: n=48; 44MF, 1PMF, 3M) were excluded from further analysis. Subsequent analysis of infradian rhythm prevalence required participants to have at least one statistically significant cycle period of more than 4 days. The infradian chronotype analysis was conducted for the NetHealth study required at least 135 days of recorded heart rate data.

**Supplementary Figure 3: NetHealth CONSORT diagram illustrating participant flow through the study analyses.** A total of 623 (n=306F) participants were observed, of whom 98 (n=44F) were excluded from analysis due to insufficient data. The remaining 525 (n=262F) participants met inclusion criteria for infradian rhythm analysis, defined as ≥ 80% data completeness and ≥ 3 months of heart rate recordings. Among these participants, 369 (n=183F) exhibited at least one statistically significant cycle period ≥ 4 days and were therefore included in infradian rhythm prevalence analysis. Infradian chronotype analysis was subsequently conducted on 353 (n=177F) participants who met the additional requirement of a minimum observed cycle period of 45 days, corresponding to ≥ 135 days of recorded data (45 periods x 3 observed cycle periods).

**Supplementary Figure 4: Soochow CONSORT diagram illustrating participant flow through the Soochow study analyses.** A total of 118 participants (91 menstruating females (MF), 12 (postmenopausal females (PMF), 15 males (M)) were observed, of whom 48 (44MF, 1PMF, 3M) were excluded from analysis due to insufficient data. The remaining 70 participants (47MF, 11PMF, 12M) met inclusion criteria for infradian rhythm analysis, defined as ≥ 80% data completeness and ≥ 3 months of heart rate recordings.

### Appendix 3: Effects of Missing Data on Analysis

The effects of missing data on the prevalence of infradian rhythms analysis were explored. There was a mean missing data segment duration of 3.2 days (SD = 3.7) (Supplementary Figure 5a); thus, to further explore the effects of restricting analysis to segments with no more than 5 consecutive days missing, infradian rhythm prevalence both for dominant cycles (Supplementary Figure 5b) and all observed cycles (Supplementary Figure 5c) were analysed and findings revealed similar results to the main analysis with weekly, monthly, 10-week, biannual, and annual rhythms commonly found. This highlights the robustness of the findings.

**Supplementary Figure 5: Effects of missing data duration on infradian rhythms.** (a) The distribution of average missing data durations, with a mean missing data segment duration of 3.24 days (SD = 3.73). Distribution of dominant (b) and all (c) multiday rhythms after restricting data analysis to segments with no more than 5 consecutive days missing. Histograms show the proportion of people with a dominant multiday rhythm at each cycle period (x-axis), with bar widths increasing from 2 days to 5 days at the 52-day mark. The y-axis on the right-hand side shows the sample size used at each cycle period. This is further split by sex in (c).

### Appendix 4: Selection of Chronotypes with Non-negative Matrix Factorisation

The cross-correlation between non-negative matrix factorisation (NMF) components revealed the highest cross-correlation values between features for three components following the trivial one component solution (Supplementary Figure 6).

**Supplementary Figure 6: Cross correlation between component groups derived from NNMF decomposition**. The plot displays cross-correlation values between component groups (clusters 1- 10), with each decomposition computed by seeding the algorithm with 10 different initial matrices.

### Appendix 5: Cycle Synchrony

**Supplementary Figure 7: Wavelet periodogram coherence between pairs of individuals.** Figure displays two metrics (Peak Similarity and Wasserstein Distance) used for assessing the coherence between pairs of individuals with ‘close’ (i.e., friends, family, significant others) and ‘not close’ (i.e., strangers) relationships.

Individuals in the cohort were required to periodically complete social Network Surveys through which they listed 20 other individuals with whom they interacted with over the previous few months. This list often contained other participants in the study. To investigate physiological synchrony in heart rate cycles, cycles were compared between those with close relationships and those without close relationships in the cohort. The overall degree of “closeness” in a relationship was determined by how the individual defined others in their network and the frequency of contact between the two individuals. Family members (including two pairs of siblings identified), romantic partners, roommates, and individuals in contact on daily and weekly bases were defined as “Close”; on the other hand, those who did not fit into these categories (i.e., strangers, acquaintances, and friends with monthly or less than monthly frequencies of contact) were considered “Not Close”.

Two techniques – Peak Similarity (PS) and Wasserstein Distance (WD) – were employed to quantify the similarity between two individuals’ periodogram peaks and overall shape. WD is a distance measurement between probability distributions, or in this case, two periodograms: a smaller WD indicates greater similarity between the periodograms. PS was determined by comparing the proportions of similar peaks within a pair. The individual with the greater number of peaks was set as a base for comparison, and the proportion of matching peaks to the other individual was calculated and regarded as the value of PS for that pair. If both individuals had the same number of peaks, the proportions and overall PS value were identical.

The results of this analysis are displayed in Supplementary Figure 7, visually depicting how those with closer relationships tended to have higher PS and lower WD values. As individuals could contribute to multiple pairwise observations within the social network, reciprocal pairs were deduplicated by retaining the first occurrence, yielding 5,007 unique undirected pairs. Generalised Estimating Equations (GEE) with a Gaussian family and exchangeable correlation structure were then applied, clustering observations by individual to account for within-individual correlation. There was a statistically significant difference in PS values between the 'Close' and 'Not Close' groups (p = 0.010). There was not a significant difference found for WD (p = 0.063), though the direction of the effect was consistent with closer pairs having more similar periodogram distributions. These results indicate that individuals in closer relationships tended to share similar underlying heart rate cycles and are thus more physiologically synchronised.

### Appendix 6: Prevalence of heart rate cycles

Overall, 49% (182/369) of people had a single dominant significant cycle and 32% (117/369) exhibited two significant cycles Supplementary Figure 8a. There was a significant drop-off in the proportion of people with over two significant cycles, with around 15% exhibiting three cycles and a further reduction of 3% exhibiting four cycles. The notable drop-off in the proportion of people exhibiting over two cycles is likely due to the inter-individual variability in dataset lengths, over which longer cycles might not be observed due to these limitations. For individuals with at least two significant cycles, those with a strongest cycle between 60-80days and 168.5-195 days commonly shared a weekly (7-day) cycle as their strongest secondary cycle (Supplementary Figure 8b). Similarly, those with a strongest cycle between 168.5-195days exhibited a strongest secondary cycle between 75.5-86days or between 99-113days.

**Supplementary Figure 8: (a) Hist/Distribution of number of cycles. (b) Hexbin visualization of the dominant two cycle periods in people with at least two cycles. Secondary cycle is the second most prominent peak in the wavelet periodogram.**

### Appendix 7: Distribution of infradian heart rate rhythms

The distribution of the most dominant multiday heart rate cycle across the population (Supplementary Figure 9) displayed similar clusters when considering all cycles across the population (Figure 2 in manuscript), with peaks in the histogram corresponding to weekly (7-day), monthly (30-day), 75-days, 6-month, and 1-year periods. Compared to the distribution of all cycles across the population, the proportion of people with a dominant shorter cycle (i.e., weekly, monthly and 75-day) appeared to be half the overall distribution whilst the longer cycles (i.e., 6-month cycles and 1 year) although still prominent, remained a similar proportion of the distribution.

**Supplementary Figure 9: Distribution of the most dominant infradian heart rate rhythm amongst individuals.** Histogram showing the proportion of people with a dominant multiday rhythm at each cycle period (x-axis), with bar widths increasing from 2 days to 5 days at the 52-day mark. The y-axis on the right-hand side shows the sample size used at each cycle period.

The distribution of the most dominant multiday heart rate cycle across the population (Supplementary Figure 10) displayed similar clusters when only using individuals daily mean HR (as provided in the publicly available NetHealth data website).

**Supplementary Figure 10: Distribution of the most dominant infradian heart rate rhythm amongst individuals using daily mean heart rate.** Histogram showing the proportion of people with a dominant multiday rhythm at each cycle period (x-axis), with bar widths increasing from 2 days to 5 days at the 52-day mark. The y-axis on the right-hand side shows the sample size used at each cycle period. This analysis was conducted using the daily mean heart rate provided through the publicly available NetHealth dataset.

### Appendix 8: Stability of multiday rhythms

**Supplementary Figure 11: Stability of multiday rhythms over time using autocorrelation**. (a) Spectrogram of one subject over 305 days. (b) Autocorrelation Pearson coefficients between average periodogram (shown in (c)) and spectrogram at each time-point where power was calculated for all frequency bins. (c) Average periodogram revealing one significant peak at approximately 12 weeks.

**Supplementary Figure 12:** Distribution of average correlation values (R) across all participants.

### Appendix 9: Environmental effects

The distribution of all cycles across the cohort was found following the regression of environmental effects (i.e., day-of-week, lunar phase and season) from the dataset (Supplementary Figure 13). The main striking difference between the distribution prior to and after regression is the diminished weekly 7-day cycle. While the marked 75-day rhythm appears more visually prominent, this cycle and all other cycles remained at a similar proportion of prevalence in the population.

**Supplementary Figure 13: Histogram of all cycles across the cohort after regressing out environmental effects (i.e., day-of-week, lunar phase, seasons).** Histogram showing the proportion of people with a dominant multiday rhythm at each cycle period (x-axis), with bar widths increasing from 2 days to 5 days at the 52-day mark. The y-axis on the right-hand side shows the sample size used at each cycle period.

**Supplementary Table 2: Number of individuals with cycles at different multiday periodicities after regressing out environmental effects (i.e., day-of-week, lunar phase, seasons).** Table reporting the breakdowns by sex for individuals whose strongest cycle and significant cycle(s) fall within each cycle-duration category (i.e., 5-9 days, 25-35 days, 8-12 weeks, 5-7 months, 11-13 months). For each category, the overall sample size is provided, reflecting the reduction in sample size for longer periodicities due to variation in length of data recordings across participants.

| Cycle Duration | Sample Size | N Strongest Cycle | % Female | % Male | N Significant Cycle | % Female | % Male |
| --- | --- | --- | --- | --- | --- | --- | --- |
| Weekly  5-9 days | 525 | 21 (4%) | 33 | 67 | 31 (6%) | 29 | 71 |
| Monthly  25-35 days | 505 | 6 (1%) | 67 | 33 | 19 (4%) | 74 | 26 |
| 10-week  8-12 weeks | 383 | 89 (23%) | 43 | 57 | 112 (29%) | 42 | 58 |
| Biannual  5-7 months | 234 | 43 (18%) | 65 | 35 | 65 (28%) | 55 | 45 |
| Annual  11-13 months | 99 | 19 (19%) | 47 | 53 | 28 (28%) | 50 | 50 |

### Appendix 10: Behavioural Effects

The behavioural effects of sleep (Supplementary Figure 14) and activity (Supplementary Figure 15) across the day-of-week, lunar and seasonal cycles were examined across the whole cohort and in individuals with significant weekly, monthly, and annual heart rate rhythms. Significant associations between daily sleep duration and both the day-of-week and seasonal cycle were observed in the whole cohort and among those with significant weekly, monthly, and annual heart rate rhythms (all p<0.0001). Further, significant associations with the lunar cycle were observed only in individuals with significant monthly heart rate rhythms (p=0.016), but not across the cohort. Similarly, significant correlations between daily activity and the day-of-week, lunar, and seasonal cycles were found both across the whole cohort (all p<0.0001) and among individuals with corresponding significant heart rate rhythms (day-of-week: p<0.0001, lunar cycle: p=0.04, seasonal cycle: p=0.01). Although these associations were significant correlations, resting heart rate was less strongly coupled to daily sleep duration and daily activity levels than daily mean heart rate (Appendix 1).

**Supplementary Figure 14: Within-individual standardised daily sleep duration across environmental factors (day-of-week, lunar and seasonal cycles).** Day-of-week (7 day) influence demonstrated in **a** & **b**, lunar (29.5 day) influence demonstrated in **c** & **d**, and seasonal (365.25 day) influence demonstrated in **e** & **f**. Daily sleep duration was first standardised within individuals and then means of the standardised duration were calculated per cycle phase (e.g., Winter, Spring, Summer, and Autumn), for each individual. The first column (**a**,**c**,**e**) plots the distributions of individuals’ means of standardised daily sleep duration values across phases of the environmental cycle, for the whole cohort (n=525 per phase, per environmental factor). The second column (**b**,**d**,**f**) also plots the distribution of individuals’ means of standardised daily sleep duration values across phases of the environmental cycle, but only for people whose strongest cycle was (**b**) weekly, defined as 5-9 days, (n=50) (**e**) monthly, defined as 25-35 days, (n=38) or (**h**) annual, defined as 11-13 months (n=22).

**Supplementary Figure 15: Within-individual standardised daily physical activity across environmental factors (day-of-week, lunar and seasonal cycles).** Day-of-week (7 day) influence demonstrated in **a** & **b**, lunar (29.5 day) influence demonstrated in **c** & **d**, and seasonal (365.25 day) influence demonstrated in **e** & **f**. Daily physical activity minutes (defined as the sum of “very active” and “fairly active” minutes per day, recorded by the smartwatch) was first standardised within individuals and then means of the standardised activity were calculated per cycle phase (e.g., Winter, Spring, Summer, and Autumn), for each individual. The first column (**a**,**c**,**e**) plots the distributions of individuals’ means of standardised daily physical activity values across phases of the environmental cycle, for the whole cohort (n=525 per phase, per environmental factor). The second column (**b**,**d**,**f**) also plots the distribution of individuals’ means of standardised daily physical activity values across phases of the environmental cycle, but only for people whose strongest cycle was (**b**) weekly, defined as 5-9 days, (n=50) (**e**) monthly, defined as 25-35 days, (n=38) or (**h**) annual, defined as 11-13 months (n=22).

### Appendix 11: Exercise and Sleep Distributions

The distribution of weekly exercise and sleep across the cohort was determined in order to generate the respective subplots in Supplementary Figure 16 To analyse the relationship between these two factors and the chronotypes, individuals in the cohort were separated into defined categories for exercise and sleep.

The average American guidelines for weekly exercise of 150-300 minutes (i.e., 2.5 to 5 hours) per week, although a National Health Interview Survey found that only 24% of American adults met these guidelines. For this cohort, it seemed that most either met the guidelines or exceeded them (as seen in Supplementary Figure 16a), suggesting these guidelines were optimal criteria to categorise weekly physical activity. Using these guidelines, three distinct categories were delineated to classify varying levels of physical activity: ‘Very Active’, ‘Moderately Active’, and ‘Less Active’. Thus, individuals’ weekly physical activity levels were designated as ‘less active’ for less than 2.5 hours, ‘moderately active’ as between 2.5-5 hours, and ‘very active’ as more than 5 hours per week.

The sleep duration distribution (Supplementary Figure 16b) lay around an average of 8 hours of sleep each night, which aligned with the National Sleep Foundation recommended 7-9 hours of sleep for young adults and is consistent with literature; thus, sleep categories for this cohort were defined as less than 8 hours for ‘short sleep’ and more than 8 hours for “long sleep”.

**Supplementary Figure 16: Distribution of weekly activity and daily sleep duration across the cohort.** Figure displays the weekly average activity (hours) on the left and the daily average sleep (hours) on the right. (a) The average weekly activity was computed as the sum of activity minutes where the activity level was ≥3.0 METs. (b) Daily sleep averages were calculated for individuals with at least 14 days of sleep data, where sleep was defined as ≥4 consecutive hours, allowing interruptions of less than one hour.

### Appendix 12: Links between chronotypes and demographics/behavioural factors

A multinomial logistic regression model was utilised to explore links between multiday chronotypes and demographics or behavioural factors. The outcomes of this model are shown in Supplementary Table 3. Note that odds ratios and corresponding 95% confidence intervals were obtained by taking the exponent of the coefficient estimates.

**Supplementary Table 3: Multinomial logistic regression outcomes**. Holding variables constant (i.e., weekly chronotype, Very Active’ activity levels, Female, and Longer Sleep), a multinomial logistic regression model is fit to the data to predict an individual’s chronotype category from their sex (male and female), physical activity level (less active, moderately active and very active), circadian chronotype (morning, intermediate and evening) and sleep duration (shorter sleep vs longer sleep). Note that the ‘definite’ and ‘moderate’ morning/evening types were combined in the circadian chronotype category. Each of these categories is listed under the ‘term’ column next to the various chronotypes (y-level column). There are six corresponding columns that describe an estimate (regression coefficient), standard error, statistic (z-value), p-value, as well as low and high confidence levels (95% confidence interval).
